# CXCR4+ cells in NPC tumor sphere have metastatic potential

**DOI:** 10.1016/j.bjorl.2026.101799

**Published:** 2026-03-27

**Authors:** Zhenwei Zhu, Jingyu Li, Jingxian Liu, Yuan He, Pei Guo, Kaitai Yao, Lin Chen

**Affiliations:** a Shenzhen Hospital of Southern Medical University, Department of Oncology, Shenzhen, P.R. China; b Southern Medical University, School of Basic Medical Sciences, Cancer Research Institute, Key Laboratory of Tumor Immunology Research, Guangzhou, P.R. China; c Southern Medical University, Zhujiang Hospital, Department of Pathology, Guangzhou, P.R. China; d University of Science and Technology of China, Division of Life Sciences and Medicine, The First Affiliated Hospital of USTC, Department of Radiation Oncology, Hefei, P.R. China; e Shenzhen Hospital of Southern Medical University, Department of Pathology, Shenzhen, P.R. China; f Sun YatSen University, The First Affiliated Hospital, Department of Pathology, Guangzhou, P.R. China

**Keywords:** Nasopharyngeal carcinoma, Metastasis, CXCR4/SDF1, Epithelial-mesenchymal transition, CD133

## Abstract

**Objective:**

Nasopharyngeal Carcinoma (NPC) tumor stem cells play a crucial role in the occurrence and development of nasopharyngeal carcinoma, but the identity and role of Cancer Stem Cell (CSC) subpopulations that drive metastasis remain unclear. This study explores the mechanisms of NPC stem cell subpopulations (CXCR4+) in the development of NPC, providing a reference for therapeutic targets for NPC.

**Methods:**

Immunohistochemical analysis of CXCR4 expression was performed in 71 NPC and 5 chronic nasopharyngitis tissues. Stemness, tumorigenicity and Epithelial-Mesenchymal Transition (EMT) profiles were established for cells in tumor spheres by flow cytometry (CD133/CXCR4), functional assays, Quantitative Reverse Transcription Polymerase Chain Reaction (RT-qPCR) and Western blotting. CXCR4+ cell metastatic potential was evaluated in a mouse NPC model.

**Results:**

CXCR4 expression correlated with NPC T and N stage. CD133+ and CXCR4+ cells were enriched in the tumor sphere. CXCR4+ subsets had enhanced self-renewal (The CXCR4-positive subgroup exhibited a 45% increase in the ability to form tumor spheres), spindle morphology and migration/invasion with upregulation of markers of EMT, Snail, Twist, Vimentin, N-cadherin, stemness, Oct4, Nanog, Sox2 and downregulation of E-cadherin. CXCR4+ tumorsphere cells potentiated metastatic lung nodules in vivo via SDF-1/CXCR4 signaling, giving a 500% increase relative to controls.

**Conclusion:**

A metastatic CSC subpopulation present in NPC express CXCR4 and have activated EMT and SDF-1/CXCR4-driven metastasis. CXCR4 may have utility as a biomarker and the SDF-1/CXCR4 axis may be a therapeutic target for NPC.

## Highlights

• CXCR4+ NPC cells show enhanced stemness, EMT, and metastasis.
• CXCR4 correlates with NPC T/N stage and promotes tumor sphere formation.
• SDF-1/CXCR4 axis drives metastasis with 500% increased lung nodules.
• CXCR4+ subset upregulates Snail, Twist, Oct4, and downregulates E-cadherin.
• CXCR4 may serve as a biomarker and therapeutic target for NPC.

## Introduction

Nasopharyngeal Carcinoma (NPC) originates in the epithelium usually of the top and sidewall of the nasopharynx. NPC incidence shows a geographical demarcation and ethnic susceptibility, being more common in South China and Southeast Asia, especially in the Guangdong and Guangxi provinces.^1^ It is usually treated by radiotherapy and chemotherapy and survival times of patients with nonmetastatic NPC have improved but 15% patients have distant metastases on presentation,^2^ resulting in a poor response to conventional first-line chemoradiotherapy and poor prognosis.^3^ Pathophysiological mechanisms involved in NPC metastasis remain poorly characterized and greater understanding may reveal novel therapeutic and prognostic approaches.

Tumor spheres are three dimensional tumor models that are enriched with cells showing properties of self-renewal and tumorigenesis or Cancer Stem Cells (CSC).^4^, ^5^, ^6^, ^7^ Thomas Brabletz et al.^8^ identified two dynamic populations of tumor stem cells: stable tumor stem cells and Metastatic Cancer Stem Cells (MCSCs) of which the latter grouping represent stem cells that acquire invasive properties via the Epithelial-Mesenchymal Transition (EMT). Snail, Twist, Zeb and other transcription factors regulate E-cadherin, N-cadherin and vimentin expression to alter tumor cell characteristics of adhesion, migration and invasion. Stemness markers, SOX2, OCT4 and Nanog, and Wnt and TGF signaling also influence the dynamics of the two tumor stem cell populations.^9^^,^^10^

A subpopulation of CD133+ and CXCR4 + CSCs have been implicated in pancreatic cancer metastasis^11^^,^^12^ and the interaction of CXCR4 with its CXCL12 (SDF-1) ligand is thought to regulate tumor proliferation, migration and angiogenesis in gliomas,^13^, ^14^, ^15^ giving evidence for the involvement of the CXCR4 marker in tumor metastasis and progression.^16^, ^17^, ^18^ CXCR4 is generally acknowledged to be a MCSC marker^11^^,^^12^^,^^19^ and CXCR4^+^ NPC cells appear to be more invasive and metastatic.^20^, ^21^, ^22^ CD133 is also an accepted NPC stem cell marker. The current study used the model of the NPC tumor sphere to take advantage of this model’s enrichment of NPC stem cells and overcome the paucity of CD133+ cells in the cultured NPC population. The expression of CXCR4 in NPC tissues and tumor spheres was measured and the role of CXCR4 + CSC subsets in metastasis investigated. The aim was to identify any subset of NPC cells responsible for metastatic activity, as distinct from tumorigenic activity, and to expose the possibility of CXCR4 signaling as a therapeutic target.

## Methods

### Cell culture

The nasopharyngeal epithelial cell-line, NP69, immortalized by SV40 large T antigen transfection while retaining some characteristics of normal nasopharyngeal epithelial cells and NPC cell lines, 6‒10B (weakly metastatic), 5–8F (strongly metastatic), CNE1 (highly differentiated), CNE2 (poorly differentiated) and Sune1 (poorly differentiated), were obtained from the Cancer Research Institute, Southern Medical University (Guangzhou, China). Cells were cultured in Roswell Park Memorial Institute (RPMI) 1640 medium supplemented with 100 units/mL penicillin and 100 μg/mL streptomycin and 10% bovine serum (Biological Industries, Kibbutz Beit-Haemek, Israel) at 37 °C in 95% air/5% CO_2_. The EBV + NPC cell-line, C666-1, was considered for inclusion, as a generous gift from Prof. GSW Tsao of University of Hong Kong.^23^ However, C666-1 cells had a low capacity for tumor sphere formation, making this cell-line unsuitable for inclusion in the current work.

### Tumor sphere culture

Cells were harvested, washed four times with PBS, digested with trypsin and suspended in serum-free medium DMEM/F12 (Biological Industries) with 2% B27 neuronal cell supplement, 20 ng/mL epidermal growth factor and 10 ng/mL basic fibroblast growth factor (all from PeproTech, Rocky Hill). 500 cells/mL were seeded into ultralow attachment 6-well plates (Corning, Inc., Corning, NY, USA) in serum-free medium, incubated for 7-days and lines drawn on the bottom of the plate to enable identification of tumor spheres with more than 50 cells and approximately 100 μm diameter under an inverted microscope.

### Quantitative Reverse Transcription Polymerase Chain Reaction (RT-qPCR)

Cell monolayers were cultured for 24 h and tumor spheres for 7-days before harvesting. RNA was isolated by RNAiso Plus Extraction Kit (TaKaRa Bio, Inc., Otsu, Japan), according to the manufacturer’s instructions, and quantified by Thermo NanoDrop 2000 (Thermo Fisher Scientific, Inc.). mRNA was converted into cDNA by reverse transcriptase reagent kit (Takara Biotechnology, Japan) and qPCR performed using RT-qPCR Master Mix reagent kit (Takara Biotechnology, Japan). Primer sequences for Oct-4, Sox2, Nanog, β-catenin, Snail, Vimentin, E-cadherin, Twist, CXCR4 and GAPDH are given in Table 1. GAPDH was used as internal control. PCR consisted of repeated cycles of denaturation at 95 °C for 2 min, followed by 42 cycles of denaturation at 95 °C for 15 s, annealing and extension at 60 °C for 30 sec and a final incubation at 72 °C for 30 sec in a Bio-Rad PCR thermocycler. RT‑qPCR data was analyzed by 2^−ΔΔCq^ method and is expressed as fold change. The primers are listed in Table 2.

**Table 1:** Clinical characteristics of 71 human NPC tissues.

Clinico-pathological characteristics	Total cases	CXCR4 negative	CXCR4 positive	CXCR4 strong positive	Positive rate	p^a^	Correlation coefficient	p^b^
Gender								
Male	45	25	8	12	44.4%	0.530	0.021	0.864
Female	26	15	4	7	42.3%			
Age								
<50	28	14	6	8	50%	0.199	−0.129	0.282
≥50	43	26	6	11	39.5%			
TNM stage^c^								
T status								
T1‒T2	38	27	5	6	28.9%	0.009	0.318	0.007
T3‒T4	33	13	7	13	60.6%			
N status								
N0‒N1	37	32	5	0	13.5%	0.000	0.634	0.000
N2‒N3	34	8	7	19	76.5%			
M status								
0	69	40	10	12		‒	‒	‒
1	2	0	0	2	100%			
Lymph node metastasis NPC tissue	11	0	3	8	100%	‒	‒	‒

a Fisher’s exact test.

b Spearman correlation analysis.

c TNM is a cancer staging system and was assessed according to the 8th edition of the UICC (International Union against Cancer); TNM, tumor-node-metastasis.

**Table 2:** Primers for RT-qPCR.

Gene		Sequence (5’-3’)
Sox2	Sense	AGAACCCCAAGATGCACAAC
	Antisense	ATGTAGGTCTGCGAGCTGGT
Nanog	Sense	CAAAGGCAAACAACCCACTT
	Antisense	ATTGTTCCAGGTCTGGTTGC
Oct	Sense	AGTGAGAGGCAACCTGGAGA
	Antisense	CAAAAACCCTGGCACAAACT
Snail	Sense	CAGTGGGAGACCTCGAGAAG
	Antisense	TCCCTCGGAACATCAGAAAC
Beta-catenin	Sense	CATTACAACTCTCCACAACC
	Antisense	CAGATAGCACCTTCAGCAC
N-cadherin	Sense	ACAGTGGCCACCTACAAAGG
	Antisense	CCGAGATGGGGTTGATAATG
Vimentin	Sense	GAGAACTTTGCCGTTGAAGC
	Antisense	GCTTCCTGTAGGTGGCAATC
Twist	Sense	CAGCGCACCCAGTCGCTGAA
	Antisense	CCAGGCCCCCTCCATCCTCC
GAPDH	Sense	GAAGGTGAAGGTCGGAGTC
	Antisense	GAAGATGGTGATGGGATTTC

RT-qPCR, Quantitative Reverse Transcription Polymerase Chain Reaction.

### Western blot analysis

Cells were lysed and proteins extracted by kit (Beyotime Biotechnology, China) and protein concentration evaluated by BCA reagent kit (Beyotime Biotechnology, China). Proteins were separated on a 10% SDS PAGE gel, transferred to PVDF membrane (Millipore, Boston, USA), membrane blocked with 3% BSA (Sigma-Aldrich) in Tris-buffered saline at room temperature for 1 h and incubated with primary antibodies raised against GAPDH (ab9485; 1:2,500), Vimentin (ab45939; 1:1,000), N-cadherin (ab18203; 1:1,000), E-cadherin (ab15148; 1:1,000) or CXCR4 (ab74012; 1:1,000, all antibodies from Abcam, USA) at room temperature for 2 h or 4 °C overnight. Membranes were washed for 4 × 10 min in TBST solution and incubated with HRP-labelled goat anti-rabbit secondary antibody (BS13278; 1:5,000; Bioworld Technology, USA) for 1 h before washing 3× with TBST solution. Immunoblots were visualized by enhanced chemiluminescence (Pierce; Thermo Fisher Scientific, USA) using GAPDH expression as internal control and analyzed via software suite (Image Lab software 3.0, Bio-Rad Laboratories, Inc.).

### Flow cytometry analysis

#### Marker detection

Cell surface CD133 was detected by anti-CD133-phycoerythrin antibody (Dilution ratio 1:10, Miltenyi Biotec GmbH, Germany) and CXCR4 by anti-CXCR4-APC (Miltenyi Biotec, Germany). Monolayer culture and tumor spheres were treated with 0.25% trypsin, fixed with 4% Paraformaldehyde (PFA) and stained with anti-CD133-PE or anti-CXCR4-APC in the dark at 4 °C for 30 min before detection by flow cytometer (Becton Dickinson) and analysis by FlowJo software suite (FlowJo LLC, Ashland, USA).

#### Cell sorting

CXCR4+ cells were isolated from monolayer cultures and tumor spheres using a magnet activated cell sorting system (cat. MSPB-6003, MACS, MagniSort®, eBioscience, USA) and anti-CXCR4-biotin antibody (1:100, eBioscience, USA), according to the manufacturer’s instructions. Isolated cells were cultured in RPMI-1640 complete medium (monolayer culture cells) and serum‑free DMEM/F12 (tumor sphere cells).

### Migration and invasion assays

Transwell filters (8-mm pore size) were either coated or not with 50 μL/well 1 mg/mL Matrigel (BD Biosciences) for use with 24-well plates. Cells at a density of 1 × 10^5^ cells/well in serum-free medium were inoculated into the top chamber and 500 μL DMEM with 10% FBS into the lower chamber for 48 h incubation. Cells were removed from the Transwell upper surface and those on the lower surface were fixed with 100% methanol, stained with 0.5% hematoxylin solution, washed and dried. Cells were counted in five random microscopic fields with a hematocytometer under an inverted microscope.

### Immunohistochemistry

76 paraffin wax-embedded specimens of NPC tissue and non-tumor tissue from patients with rhinitis were obtained from the The Zhujiang Hospital of Southern Medical University. No patient had received chemoradiotherapy prior to biopsy. The 71 NPC samples included 60 primary tumors and 11 secondary lymph node tumors. Two cases had distant metastasis (1700065: left submaxillary; 1701840: right eyebrow arch). 5-μm pathological tissue sections were baked at 72 °C for 1 h, dewaxed in xylene 3 times for 10 min each and treated with an ethanol gradient (100% → 95% → 80% → 70%). Slides were heated in citric acid buffer, pH 6.0, for 30 min, incubated with 3% hydrogen peroxide solution for 20 min to inhibit endogenous peroxidase and incubated with primary antibodies, anti-CXCR4 (1:100, ab74012, Abcam) and anti-CK (1:200, #54135S, CST), at 4 °C overnight. Secondary antibody, biotinylated goat anti-rabbit IgG (LSAB, IHC detection system kit, IHC001, Bioss), was added for 1 h at room temperature, sections washed in PBS and incubated with HRP conjugate for 20 min. before visualization with 30‒50 μL DAB solution (Bioss) and staining with hematoxylin. Sections were inspected under an inverted microscope.

### Tail vein metastatic surrogate assay

5 × 10^5^ aliquots 5‒8F CXCR4-positive and CXCR4-negative cells were injected into the caudal vein of male BALB/c nude mice (4-weeks, 16 grams). Mice were divided into four groups (n = 3): CXCR4+; CXCR4-; CXCR4+ treated with CXCR4 inhibitor, AMD3100; CXCR- with AMD3100. Mice treated with AMD3100 were given 2.5 μg/g AMD3100 every two days by intraperitoneal injection for a total of 10 times. Animals were housed in specific pathogen-free conditions at a constant 20 °C temperature, 50% humidity and 12 -h light/dark cycle in the animal facility, Southern Medical University. Fluorescence intensity of metastatic lesions was monitored using In Vivo Imaging System (IVIS). Mice were sacrificed 4-weeks after inoculation. All animal experiments were performed in accordance with the Guide for the Use and Care of Laboratory Animals and ethical approval was granted by the Experimental Animal Ethics Committee, Southern Medical University (nº 2022-0236).

### Statistical analysis

Data are presented as mean ± SD. Tumor/metastasis formation rates in the mouse model were compared by Fisher’s exact test. Intergroup differences were analyzed by Student’s *t*-test. Categorical variables of M status, N status, sex and location were analyzed by Fisher’s exact test. Ranked variables of T-status and Tumor-Node-Metastasis (TNM) staging and randomly permuting samples of high versus low CXCR4+ cell content were analyzed by permutation test. All statistical analyses were performed using SPSS 17.0 software suite (SPSS, Inc., Illinois). All tests were 2-tailed and a p-value < 0.05 was considered to indicate statistical significance. All data is the result of at least 3 independent experiments.

## Results

### CXCR4 expression in NPC tissues

**Fig. 1:**
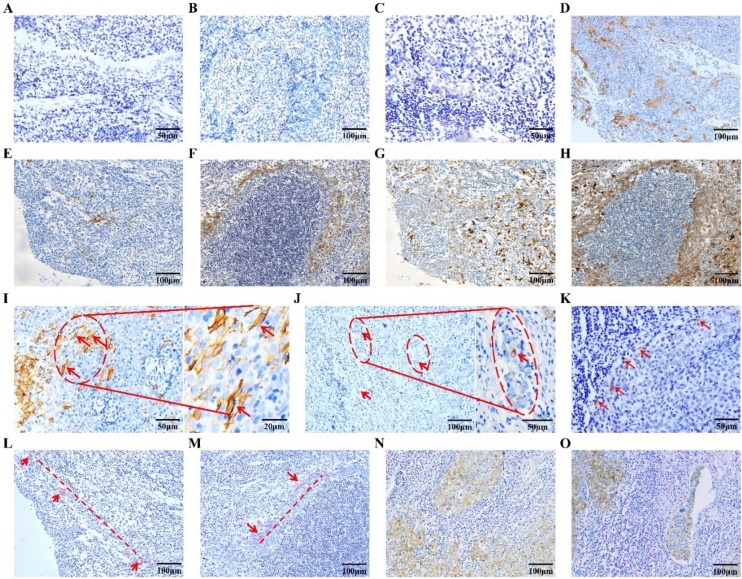
Spindle-shaped CXCR4+ cells in NPC tumor and rhinitis non-tumor tissues. (A) CXCR4 expression (non-detectable) in nasopharyngeal rhinitis tissue; (B‒D) CXCR4 expression in nasopharyngeal carcinoma tissues, (B) Non-deectable, (C) Weakly positive (≤10% of all cancer cells) and (D) Strongly positive (≥10% of all cancer cells); (E‒F) Immunohistochemical staining of CXCR4 in nasopharyngeal primary carcinoma tissues and secondary lymph node metastases; (G‒H) Pan-CK immunohistochemical staining in serial sections of nasopharyngeal primary carcinoma tissues and secondary lymph node metastases. Overlapping expression of CXCR4 and PAN-CK can be seen (1E and 1G; 1F and 1H). Most CXCR4+ cells were also CK+; (I) Cell membrane and cytosolic expression of CXCR4 in nasopharyngeal carcinoma cells. CXCR4+ cells were usually spindle-shaped; (J) A single or small number of CXCR4+ cells may be seen in the micrometastatic foci of lymph node metastatic cancer tissues; (K) CXCR4+ cells can be seen in tight clusters at the edges of tumor tissues; (L‒M) A line of CXCR4+ NPC cells may be seen, perhaps indicating migratory activity (red line); (N) Most metastatic NPC cells in lymph node tissue expressed CXCR4; (O) CXCR4+ NPC cells in the vasculature.

Immunohistochemical staining of 71 NPC (60 primary plus 11 lymph node secondary) and 5 non-tumor rhinitis tissues (see Table 1 for clinical details) was performed. CXCR4 expression was non-detectable in rhinitis tissues (Fig. 1A) but present in many of the 71 NPC tissues. Expression ranged from non-detectable (Fig. 1B), through weakly positive when expressed by ≤10% tumor cells (Fig. 1C) to strongly positive when expressed by ≥10% tumor cells (Fig. 1D). Pan-CK staining to show tumors of epithelial origin partially overlapped with CXCR4 expression, confirming that CXCR4+ cells were NPC cells (Fig. 1E‒H). CXCR4 expression correlated with T-stage (*r* = 0.318, p<0.01) and N stage (*r* = 0.634, p<0.001) with higher expression at stage T3‒4 than T1‒2 group (60.6% vs. 28.9%) and at stage N2‒3 than N0‒1 (76.5% vs. 13.5%, Table 1). CXCR4 showed strong positive expression in the primary pathological specimens from the two patients who had distant metastases (1700065: left submandibular; 1701840: right eyebrow arch). The marker was also expressed in all the secondary lymph node tumors with 8/11 showing strong positive expression (results not shown). The results suggest a link between CXCR4 expression and NPC metastasis.

CXCR4+ cells generally had spindle-shaped morphology with both cell membrane and cytoplasmic marker expression (Fig. 1I and 1I-enlarged). CXCR4-positive cells were sometimes tightly clustered at the edge of tumor tissues (Fig. 1K) and tended to be distributed along the invasion path (Fig. 1L and 1 M). Most lymph node metastatic cells were CXCR4+, particularly among cells within invasive regions (Fig. 1N), and a single CXCR4-positive cell could be seen in the micrometastatic lesion (Fig. 1J). CXCR4+ tumor cells could also be seen in vascular tissue (Fig. 1O), indicating a role for CXCR4+ tumor cells in NPC metastasis, vascular invasion and distant lesion formation.

### CXCR4 expression correlated with metastasis and with loss of differentiated characteristics

**Fig. 2:**
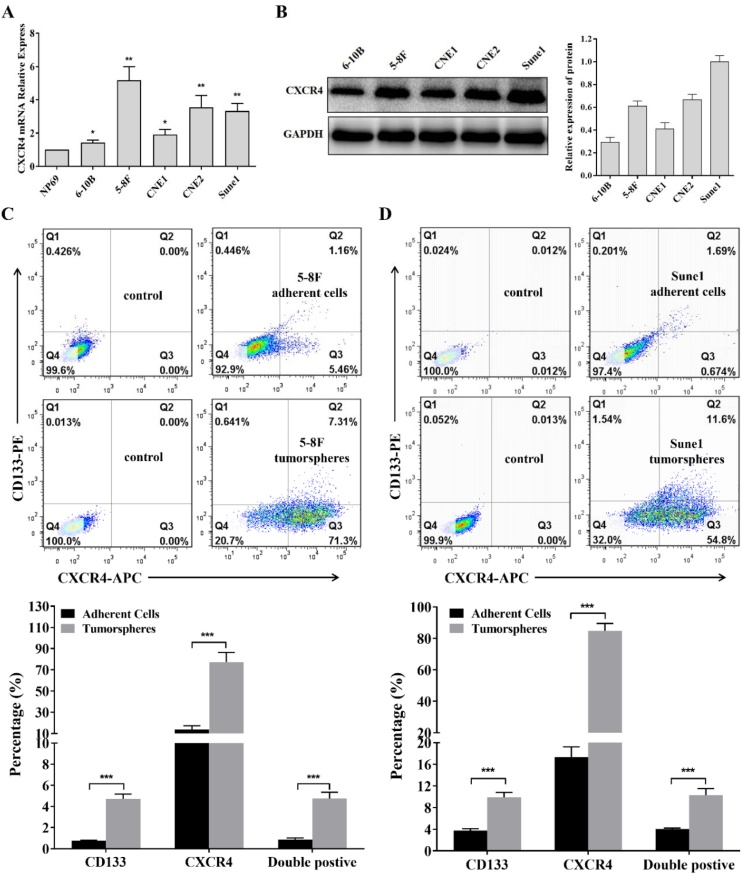
CXCR4 and CD133 expression in nasopharyngeal carcinoma cells. (A) RT-qPCR assays of CXCR4 mRNA in NPC cell-lines and NP69 control; (B) Western blotting to show CXCR4 protein in NPC cell-lines; (C) Percentage of CD133+ ((5-8F Q1 + Q2) - (control Q1 + Q2)) and CXCR4+ ((5-8F Q3 + Q4) - (control Q3 + Q4)) 5-8F adherent cells and tumor sphere cells. Most CD133+ cells were also CXCR4 + . Flow cytometry outputs are shown to the left with quantitative analysis to the right; (D) Percentage of CD133+ ((Sune1 Q1 + Q2) - (control Q1 + Q2)) and CXCR4+ ((Sune1 Q3 + Q4) - (control Q3 + Q4)) Sune1 adherent cells and tumor sphere cells. Most CD133+ cells were also CXCR4 + . Flow cytometry outputs are shown to the left with quantitative analysis to the right. Bars represent means ± SD of three independent experiments; * p < 0.05, ** p < 0.01, *** p < 0.001. RT-qPCR, Quantitative Reverse Transcription Polymerase Chain Reaction; NPC, Nasopharyngeal Carcinoma.

All NPC cell-lines tested had higher CXCR4 expression than the immortalized nasopharyngeal NP69 cell line and mRNA and protein levels were higher in 5‒8F, CNE2 and SUNE1 than in 6‒10B or CNE1 (Fig. 2A and 2B). These data are consistent with the findings from the patient samples and show that CXCR4 expression was higher in highly metastatic or poorly differentiated cells, supporting the view that CXCR4 is involved in NPC metastasis and differentiation.

### Tumor spheres show enrichment of CD133+ and CXCR4+ cells

Flow cytometry detection of cells expressing CXCR4 and CD133 showed adherent 5‒8F cells had 1.18% CXCR4+ and 6.62% CD133+ cells but tumor spheres formed from 5‒8F cells were 7.938% CXCR4+ and 78.61% CD133 + . Double-positive staining cells accounted for 1.16% of the adherent population and 7.31% of the tumor sphere population (Fig. 2C). Sune1 cells had values of 1.855% CD133+ and 2.34% CXCR4+ in the adherent population, 13.075% CD133+ and 66.387% CXCR4+ in tumor spheres and double-positive staining cells accounted for 1.678% of the adherent population and 11.587% of the tumor sphere population (Fig. 2D). A greater proportion of 5‒8F and Sune1 tumor sphere cells were CD133+ and CXCR4+ than adherent cells and most CD133-positive cells also expressed CXCR4. The data is consistent with the view that stem cells and CXCR4+ cells were enriched in tumor spheres.

### Spindle-shaped CXCR4+ cells had greater potential to form tumor spheres than CXCR4-cells

**Fig. 3:**
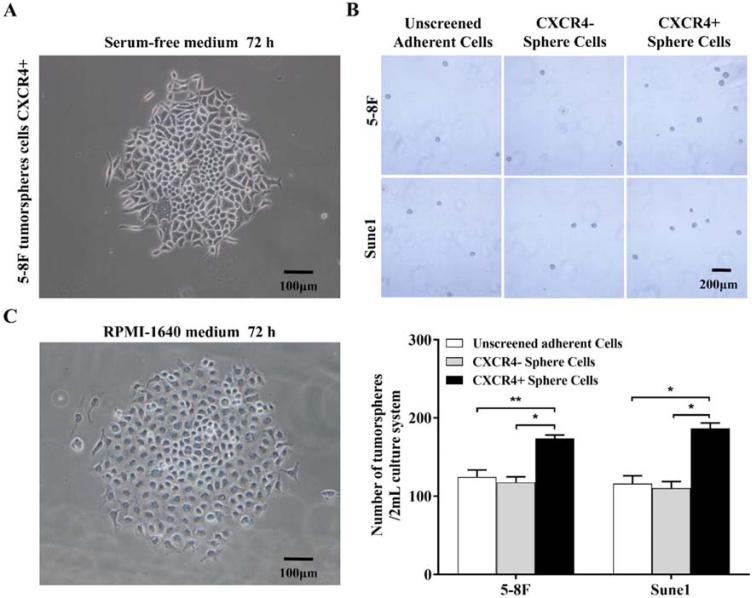
Morphological characteristics of CXCR4- and CXCR4+ tumor spheres. (A) Fusiform CXCR4+ tumor sphere cells after culture in serum-free medium; (B) Differentiated CXCR4+ tumor sphere cells after culture in complete medium; (C) Tumor sphere formation by CXCR4+ and CXCR4- cells isolated from tumor spheres cells. Microscopic images are shown on the left and quantitative analysis on the right. Bars represent means ± SD of three independent experiments. * p < 0.05, ** p < 0.01.

5‒8F CXCR4+ cells were isolated from tumor spheres by magnetic beads and cultured in serum-free or complete medium for 72 h. Most cells cultured in serum-free medium were fusiform with some polygonal cells (Fig. 3A) but those cultured in complete medium were almost entirely polygonal with characteristics of tumor cells (Fig. 3B). 5‒8F CXCR4+ cells isolated from the tumor sphere had greater tumor sphere-forming potential than CXCR4- tumor sphere or adherent cells which showed equivalent rates of tumor sphere formation (Fig. 3C). Similar results were obtained with Sune1 cells (Fig. 3C).

### CXCR4+ tumor sphere cells showed enhanced migration and invasion in vitro

**Fig. 4:**
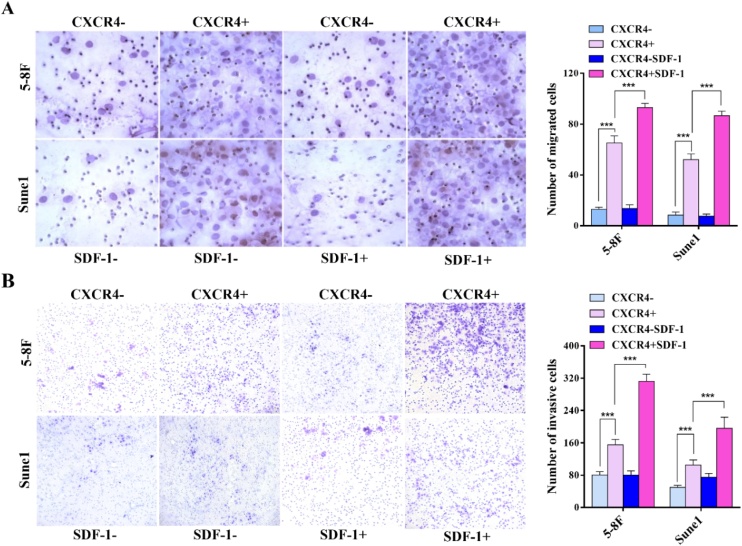
Migration and invasion of CXCR4+ and CXCR4- tumor sphere cells and the effect of SDF-1. (A) Migration of CXCR4+ and CXCR4- tumor sphere 5‒8F and Sune1 cells in the presence and absence of the CXCR4 ligand, SDF-1. Representative images are shown on the left with quantitative analysis on the right; (B) Invasion of CXCR4+ and CXCR4- tumor sphere 5‒8F and Sune1 cells in the presence and absence of the CXCR4 ligand, SDF-1. Representative images are shown on the left with quantitative analysis on the right. Bars represent means ± SD of three independent experiments. * p < 0.05, ** p < 0.01, *** p < 0.001.

5‒8F and Sune1 CXCR4+ cells isolated from the tumor sphere had higher rates of migration and invasion in Transwell assays than equivalent CXCR- cells. Treatment with the CXCR4 ligand, SDF-1, enhanced migration and invasion by CXCR4+ cells but not by CXCR- cells (Figs. 4A and 4B). The data imply the involvement of the SDF-1/CXCR4 axis in migration and invasion by 5‒8F and SUNE1 cells.

### CXCR4+ NPC stem cells were more strongly metastatic than CXCR4- cells

**Fig. 5:**
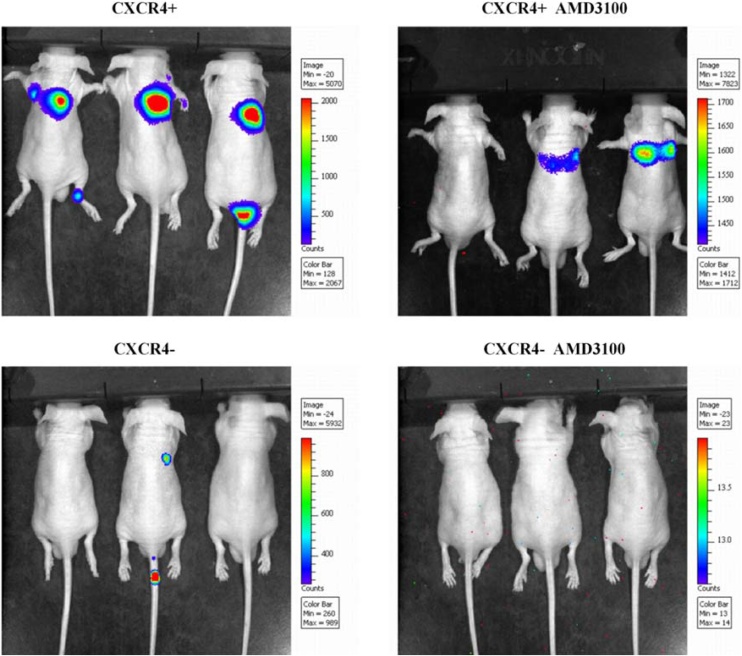
Metastasis formation from 5‒8F cells in a mouse model of NPC. IVIS images show the formation of metastases (colored areas) from inoculation with 5‒8F NPC tumor sphere cells. Inoculated cells were CXCR4+ or CXCR4-. The CXCR4 inhibitor, AMD1300, was administered, where indicated. NPC, Nasopharyngeal carcinoma; IVIS, In Vivo Imaging System.

in vivo treatment of mice with 5‒8F tumor spheres showed a higher rate of metastasis formation from CXCR4+ than from CXCR4- cells (Fig. 5). Metastasis formation was reduced when the CXCR4 inhibitor, AMD3100, was used. These data are consistent with the view that CXCR4+ tumor stem cells are more strongly metastatic and support the involvement of CXCR4 signaling in metastasis formation.

### CXCR4 influenced NPC cell EMT

**Fig. 6:**
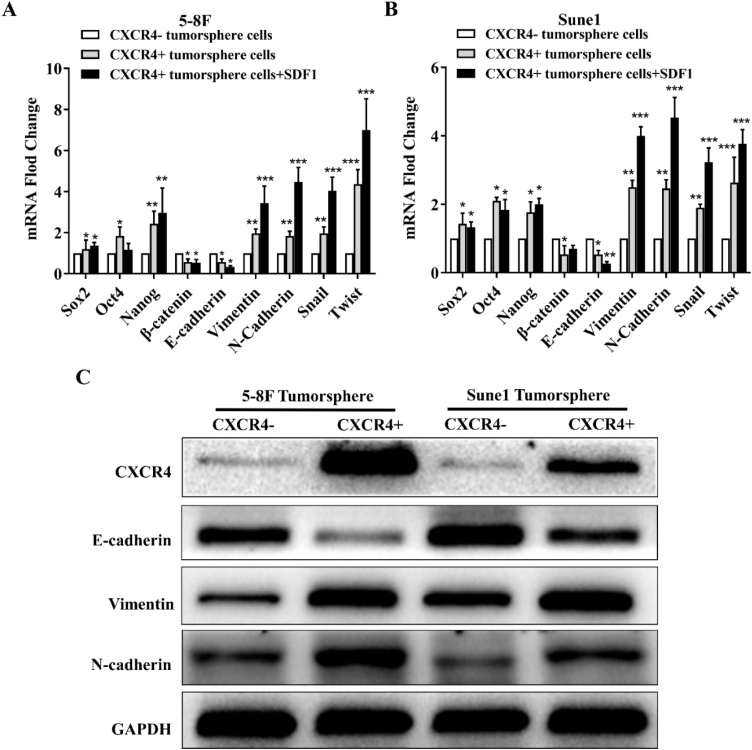
Expression of markers of stemness and of the EMT in 5‒8F and Sune1 cells. (A) Fold change in expression of mRNA of stemness genes, Oct4, Nanog and Sox2, EMT genes, Snail and Twist, mesenchymal marker genes, vimentin and N-cadherin, and of epithelial marker, E-cadherin in 5‒8F cells. Changes in mRNA expression were calculated relative to GAPDH; (B) Fold change in expression of mRNA of stemness genes, Oct4, Nanog and Sox2, EMT genes, Snail and Twist genes, mesenchymal markers, vimentin and N-cadherin, and of epithelial marker, E-cadherin in Sune1 cells. Changes in mRNA expression were calculated relative to GAPDH; (C) Western blot image to show expression of CXCR4, E-cadherin, vimentin, N-cadherin and GAPDH protein in 5‒8F and Sune1 CXCR4+ and CXCR4- tumor sphere cells. Bars represent means ± SD of three independent experiments. * p < 0.05, ** p < 0.01, *** p < 0.001. EMT, Epithelial-mesenchymal transition.

Measurement of expression of genes involved in stemness and the EMT showed higher expression of Oct4, Nanog and Sox2 stemness genes and of Snail and Twist EMT genes in CXCR4+ tumor sphere cells compared with CXCR4- counterparts (Fig. 6). In addition, CXCR4+ cells had increased expression of the mesenchymal markers, vimentin and N-cadherin, and decreased expression of the epithelial marker, E-cadherin. The data indicate an association between CXCR4 expression and activity and the acquisition of stemness characteristics plus the promotion of the EMT in NPC tumor sphere cells.

## Discussion

Distant metastasis is a major cause of NPC mortality and tumor stem cells, particularly transplantable metastatic stem cells, are thought to account for drug resistance, metastasis and recurrence.^6^, ^7^, ^8^ Baccelli et al.^24^ reported the presence of Metastatic tumor Initiation Cells (MICs) among the population of circulating tumor cells in patients with primary breast cancer and concluded that these cells were responsible for metastases in bone, lung and liver. MICs were EPCAM(+), CD44(+), CD47(+) and MET(+). In addition, a population of CD133+ CXCR4+ CSCs were held accountable for pancreatic cancer metastasis.^12^ The discovery of distinctive characteristics of a subpopulation of tumor cells with metastatic potential raise the possibility of targeting these cells to block metastasis and prolong patient survival. However, the involvement of metastatic stem cells in NPC metastasis had not been clarified.

The CXCR4+ tumor sphere cells of the current study had greater metastatic potential than CXCR4- cells and tumor sphere cells expressed the stem cell marker, CD133,^25^ at a higher level than equivalent adherent cells. Previous studies have indicated the involvement of CXCR4 in granulocyte migration during the inflammatory response, embryonic development, hematopoietic stem cell migration and homing.^26^ The SDF1/CXCR4 axis has also been implicated in cell migration, invasion, metastatic tumor formation and prognosis in a variety of tumors,^27^ including NPC.^28^, ^29^, ^30^ Indeed, SDF-1/CXCR4 blockade reduced liver metastasis from colon cancer.^31^ These observations raised the possibility of the involvement of CXCR4 in NPC stem cell migration and metastasis.

Tumor metastasis encompasses the processes of adjacent organ invasion, spread to the vasculature, circulation to target site and invasion of distant tissue and requires the acquisition of the requisite characteristics by metastatic tumor cells.^32^ Metastases are thought to originate from the expansion of a single tumor cell.^33^^,^^34^ It is noteworthy that a sample of metastatic lymph node tissue inspected during the current study contained a single CXCR4+ cell in the middle of a small mass of tumor cells. We speculate that the CXCR4+ cell migrated from the primary lesion to the lymph node and expanded to form the secondary tumor. The current findings regarding CXCR4+ cells are consistent with previously reported characteristics of metastatic tumor stem cells, including properties of accelerated EMT, migration and colonization of distant lesions.^35^

Tumor cells acquire a property of stemness through the EMT^36^, ^37^, ^38^ and this process continues to be involved in the transformation of CSCs into metastatic tumor stem cells.^39^ The current findings support the view that the chemokine receptor, CXCR4, may stimulate Snail and Twist signaling to influence the EMT. This action of CXCR4 would explain the involvement of CXCR4+ NPC cells in tumor sphere formation in vitro and in metastasis in vivo in the current study.

Although tumor sphere culture gives an in vitro model for nasopharyngeal carcinoma stem cell research, it has significant limitations for analysis of stemness characteristics due to differences between the artificial microenvironment and the in vivo niche. First, lack of microenvironmental signals distorts stemness phenotypes and there is dysregulated hypoxia tension. Physiological hypoxia (<2% O_2_) in the stem cell niche of solid tumors maintains stemness by stabilizing HIF-1α whereas culture of spheres at 20% O_2_ inhibits this pathway. Stemness factors, such as OCT4 and Nanog, are downregulated by >60% as a result,^40^ glycolysis-oxidative metabolism reprogramming reduced, and a distorted phenotypic assessment of radiotherapy resistance produced. TGF-β, IL-6 and Wnt ligands normally secreted by stromal tumor-associated fibroblasts (CAFs) are completely absent which reduces EMT stimulation and activation of stemness pathways, such as Wnt/β-catenin, compared with the in vivo state.^41^ Second, serial passage in vitro leads to an exponential decline in the core stem cell subpopulation, such as CD133+,^42^ which may represent <2% cells at P3. Stem cells in vivo would normally maintain homeostasis by asymmetric division. These changes represent a form of adaptive evolution under artificial selection pressure which does not reflect in vivo heterogeneity. Third, spheroid cells require 10^3^‒10^4^ cells to form tumors in immunodeficient mice but in situ recurrence in patients may be driven by a single circulating tumor stem cell. Thus, the spheroid model may lead to the underestimation of tumorigenic potential of in vivo stem cells. The tumor sphere model is a valuable initial screening tool for the study of stemness but its detachment from the tumor niche requires cautious data interpretation and cross-validation by Patient-Derived Organoids (PDOs) or orthotopic transplantation models to make conclusions more clinically relevant.

## Conclusions

The targeting of metastatic stem cells has the potential to block the formation of metastatic tumors. CXCR4+ cells from NPC tumor spheres had the characteristics of metastatic tumor stem cells and treatment with the CXCR4 inhibitor, ADM3100, reduced metastasis formation in the NPC animal model used in the current work. Targeting of the SDF-1/CXCR4 axis may reduce the migration of nasopharyngeal CSCs and inhibit metastasis. CXCR4 may be a therapeutic target for NPC treatment.

## ORCID ID

Zhenwei Zhu: 0009-0007-0956-4194

Jingyu Li: 0000-0002-3405-650X

Jingxian Liu: 0000-0001-9702-1876

Yuan He: 0000-0002-6461-5341

Pei Guo: 0009-0008-2221-9798

Kaitai Yao: 0000-0002-0645-0243

Lin Chen: 0000-0002-7552-8673

## CRediT authorship contribution statement

Zhenwei Zhu: Visualization; Formal analysis; Funding acquisition; Writing-review & editing.

Jingyu Li: Visualization; Resources.

Jingxian Liu: Data curation; Methodology.

Yuan He: Validation; Methodology.

Pei Guo: Validation; Investigation.

Kaitai Yao: Project administration; Supervision; Writing-review & editing.

Lin Chen: Conceptualization; Project administration; Writing-original draft, Writing-review & editing; Funding acquisition.

All authors read and approved the final manuscript.

## Funding

This work was supported by the National Natural Science Foundation of China (nº 82103504), the Medical Science and Technology Research Foundation of Guangdong Province (nº A2020507); the Basic Research Project of Science and Technology Plan of Bao'an District of Shenzhen City (nº 2018JD240); the Seedling Program of Shenzhen Hospital, Southern Medical University (nº 2018MM10); and the Sanming Project of Medicine in Shenzhen (nº SZSM201612023).

## Ethics approval

This study was performed in line with the principles of the Declaration of Helsinki. Approval was granted by the Ethics Committee of The Zhujiang Hospital of Southern Medical University (nº 2022-028). Informed consent was obtained from all subjects and/or their legal guardian(s). All animal experiments were performed in accordance with the Guide for the Use and Care of Laboratory Animals and ethical approval was granted by the Experimental Animal Ethics Committee of Shenzhen Hospital of Southern Medical University (nº 2022-0236).

## Data availability statement

The datasets and materials used in the study are available from the corresponding author.

## Declaration of competing interest

The authors declare no have conflicts of interest.
